# Revalidation of *Mazama rufa* (Illiger 1815) (Artiodactyla: Cervidae) as a Distinct Species out of the Complex *Mazama americana* (Erxleben 1777)

**DOI:** 10.3389/fgene.2021.742870

**Published:** 2021-12-14

**Authors:** Pedro H. F. Peres, Douglas J. Luduvério, Agda Maria Bernegossi, David J. Galindo, Guilherme B. Nascimento, Márcio L. Oliveira, Eluzai Dinai Pinto Sandoval, Miluse Vozdova, Svatava Kubickova, Halina Cernohorska, José Maurício Barbanti Duarte

**Affiliations:** ^1^ Deer Research and Conservation Center (NUPECCE), São Paulo State University (UNESP), Jaboticabal, Brazil; ^2^ Faculty of Veterinary Medicine, National University of San Marcos (UNMSM), Lima, Peru; ^3^ Medical School, University Center of Adamantina (UniFAI), Jaboticabal, Brazil; ^4^ Veterinary Research Institute, Brno, Czech Republic

**Keywords:** Odocoileini, GMYC, bayesian phylogenetic inference, non-invasive sampling, scat detection dog, cytotaxonomy, molecular cytogenetics

## Abstract

The red brocket deer *Mazama americana* Erxleben, 1777 is considered a polyphyletic complex of cryptic species with wide chromosomal divergence. Evidence indicates that the observed chromosomal divergences result in reproductive isolation. The description of a neotype for *M. americana* allowed its genetic characterization and represented a comparative basis to resolve the taxonomic uncertainties of the group. Thus, we designated a neotype for the synonym *Mazama rufa* Illiger, 1815 and tested its recognition as a distinct species from the *M. americana* complex with the analysis of morphological, cytogenetic and molecular data. We also evaluated its distribution by sampling fecal DNA in the wild. Morphological data from craniometry and body biometry indicated an overlap of quantitative measurements between *M. rufa* and the entire *M. americana* complex. The phylogenetic hypothesis obtained through mtDNA confirmed the reciprocal monophyly relationship between *M. americana* and *M. rufa*, and both were identified as distinct molecular operational taxonomic units by the General Mixed Yule Coalescent species delimitation analysis. Finally, classic cytogenetic data and fluorescence in situ hybridization with whole chromosome painting probes showed *M. rufa* with a karyotype of 2n = 52, FN = 56. Comparative analysis indicate that at least fifteen rearrangements separate *M. rufa* and *M. americana* (*sensu stricto*) karyotypes, which confirmed their substantial chromosomal divergence. This divergence should represent an important reproductive barrier and allow its characterization as a distinct and valid species. Genetic analysis of fecal samples demonstrated a wide distribution of *M. rufa* in the South American continent through the Atlantic Forest, Cerrado and south region of Amazon. Thus, we conclude for the revalidation of *M. rufa* as a distinct species under the concept of biological isolation, with its karyotype as the main diagnostic character. The present work serves as a basis for the taxonomic review of the *M. americana* complex, which should be mainly based on cytogenetic characterization and directed towards a better sampling of the Amazon region, the evaluation of available names in the species synonymy and a multi-locus phylogenetic analysis.

## Introduction

The genus *Mazama* Rafinesque, 1817 is the most diversified of the tribe Odocoileini with nine species of medium-sized (10–65 kg), solitary forest deer with spiked antlers, and elusive behavior (Weber and Gonzalez, 2003; Merino and Rossi, 2010; Gutiérrez et al., 2015). The taxonomy within *Mazama* was historically based on morphological data and discordant arrangements, with 2–11 species considered for the genus (Allen, 1915; Cabrera, 1960; Czernay, 1987). In this context, the red brocket deer *Mazama americana* (Erxleben, 1777), type species of the genus, had its delimitation varying depending on the study (Allen, 1915; Cabrera, 1960; Czernay, 1987) and there are currently 20 names considered synonymous (Merino and Rossi, 2010). Nevertheless, all taxonomic reviews were based on morphological data, and such characters are not informative in uncovering *Mazama* species diversity (Duarte et al., 2008; Cifuentes-Rincón et al., 2020; González and Duarte, 2020). The retention of a morphological pattern among genetic lineages within the red brocket complex does not allow their differentiation and represents a challenge in the taxonomy of the group (Cifuentes-Rincón et al., 2020).

**FIGURE 1:**
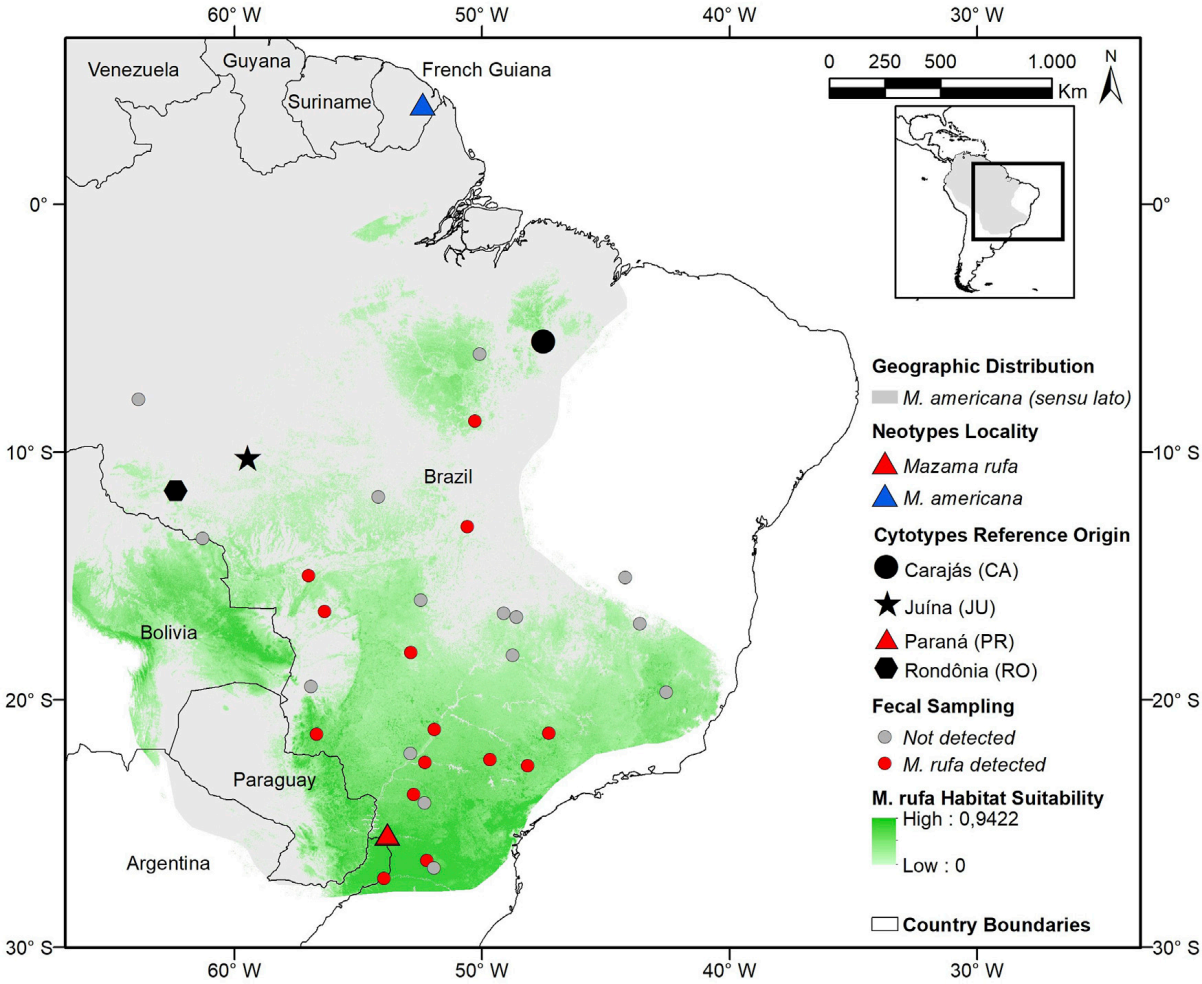
Distribution modelling (maximum entropy model, AUC = 0.908, SD 0.018, *p* < 0.001) for the red brocket deer *Mazama rufa* (Illiger, 1815) compared to the IUCN geographic distribution of *M. americana* (*sensu lato*), cytotypes reference regions, type localities, and fecal sampling sites.

The red brocket deer *M. americana* was identified as a complex of cryptic species because it does not represent a monophyletic group and presents great karyotypic diversity (Duarte et al., 2008; Abril et al., 2010; Gutiérrez et al., 2017; Heckeberg, 2020). Two species previously classified as *M. america*na, *Mazama temama* (Kerr, 1972) and *Mazama bororo* (Duarte and Jorge, 1996), have already been recognized as distinct species based on their extreme chromosomal differences (Jorge and Benirsche, 1977; Duarte and Jorge, 2003). The cytogenetic evaluation carried out by Duarte and Jorge (1996) was the first study to reveal the cryptic diversity of *M. americana* when describing chromosomal variants in Brazil. After that two distinct chromosomal lineages were identified for the species and several cytotypes (geographically established karyotypes) were described (Abril et al., 2010). One of these main lineages has a low chromosome number (2n = 42–45) and is located in the western Amazon. It is composed of cytotypes Rondônia (RO; 2n = 42/43; FN = 46) and Juína (JU; 2n = 44/45; FN = 48). The other main lineage presents a high chromosome number (2n = 49–53) and occurs in the eastern region of the Amazon and in the Atlantic Forest. This lineage is composed of the cytotypes Paraná (PR; 2n = 52/53; FN = 56), Carajás (CA; 2n = 50/51; FN = 54), Santarém (SA; 2n = 50/51; FN = 56) and Jari (JA; 2n = 48/49; FN = 56) (Abril et al., 2010; Figure 1).

Captive breeding studies showed that hybrid progeny between animals with high chromosomal divergence showed total infertility, or a high degree of subfertility due to flaws in both male and female gametogenesis (Cursino et al., 2014; Salviano et al., 2017). Subfertility was also observed, to a lesser degree, among less divergent cytotypes (Cursino et al., 2014; Salviano et al., 2017). The reproductive fitness of these same hybrids, however, must be severely aggravated by the chromosomal imbalance observed in sperm (Galindo et al., 2021). Thus, in a conservative taxonomic approach, the greatest chromosomal divergence among populations (>3 chromosome pairs) represented a post-zygotic reproductive barrier. This indicates the presence of two species in the *M. americana* complex correspondent to the two described lineages (Abril et al., 2010; Cursino et al., 2014; Salviano et al., 2017; Carranza et al., 2018). In a less conservative taxonomic approach, the subfertility observed among populations with a minor divergence (1 or 2 chromosome pairs) would already represent isolation, leading to the hypothesis that each cytotype could correspond to a distinct species (Abril et al., 2010; Cursino et al., 2014; Salviano et al., 2017; Carranza et al., 2018; Galindo et al., 2021).

Cytogenetic information is supported by molecular phylogeny data, indicating that the *M. americana* complex is not monophyletic (Duarte et al., 2008; Heckeberg et al., 2016; Gutiérrez et al., 2017; Cifuentes-Rincón et al., 2020; Heckeberg, 2020). The two chromosomal lineages were recovered in distinct well-supported clades (Abril et al., 2010), and clustered among other species (Cifuentes-Rincón et al., 2020). The lineage with the higher diploid number was recovered in a proper clade and the one with the lower diploid number was also monophyletic and was recovered as a sister group of *M. bororo* (Cifuentes-Rincón et al., 2020). Nevertheless, the reciprocal monophyly among less divergent cytotypes within each lineage (Paraná x Carajás and Juína x Rondônia) was not properly tested (Abril et al., 2010; Cifuentes-Rincón et al., 2020).

Given that no type specimen was never indicated for *M. americana* and the morphological information is not informative for the taxon, a neotype for the species was designated and cytogenetic analyses were conducted (Cifuentes-Rincón et al., 2020). The animal presented a distinct karyotype (2n = 45 and FN = 50), divergent from all cytotypes known so far. This finding raised the need to review the taxonomic status of names currently considered synonymous of *M. americana* towards the different genetic lineages observed for the species (Cifuentes-Rincón et al., 2020). The oldest available name in the timeline of the *M. americana* synonymy is *Cervus rufus* Illiger 1815 (Merino and Rossi, 2010). This species was originally described by Azara (1801) in Paraguay as the *guazupitá* deer and should correspond to the geographic occurrence of Paraná cytotype in the south of *M. americana* distribution. Its formal description was made by Illiger (1815) based on the morphological description by Azara (1801), and later transferred by Lydekker (1898) to the genus *Mazama,* which resulted in the binomial *Mazama rufa*.

Most of the mammals described by Félix de Azara did not have a specimen sent to Europe, and from those who had, few survived to serve as type material (Hershkovitz, 1989; Voss et al., 2009; Pereira, 2013). The designation of neotypes from species described from Azara’s observations has been common in literature and were essential in the organization of taxonomic nomenclature of different taxa as the case of oryzomyine rodents (Musser et al., 1998), opossums from the genus *Thylamys* (Voss et al., 2009) and felids from the genus *Leopardus* (Nascimento et al., 2020). Therefore, the indication of a neotype to anchor the name *M. rufa* is essential to clarify its taxonomic status concerning the *M. americana* complex. In particular, the taxonomic review of *Mazama* would be favored with the collection of present topotypes that provides living tissue for cytogenetic analysis. This would allow us to verify its correlation with the Paraná cytotype, the neotype specimen and elucidate the species delimitation towards *M. americana* complex.

Thus, the present study sought to 1) collect a specimen to be indicated as a neotype of *Mazama rufa*; 2) perform a morphological and cytogenetic comparison between *M. rufa* and the newly described *M. americana* neotype; 3) elucidate *M. rufa* species delimitation towards a set of specimens from the *M. americana* complex (*sensu lato*) and other *Mazama* species; 4) Estimate the geographic distribution of *M. rufa* and its potential conservation status. These efforts were based on an integrative taxonomic approach that considered the general Lineage Species Concept (De Queiroz, 2007), and the operational biological (Mayr, 1942) and phylogenetic (Cracraft, 1983) concepts based on morphological, cytogenetic and molecular data.

## Materials and Methods

### Samples

#### Type Locality and Neotype Collection

The description of *Mazama rufa* (Illiger, 1815) did not indicate nor deposit a holotype and there were no details about the collection site (Azara, 1801). The studies of Azara took place in Paraguay, along the Paraguay River, located in the Río La Plata Basin (Pereira, 2013). Two unsuccessful scientific expeditions were carried out in 2016 to collect the neotype in the region of Asunción, urban reference on where the naturalist was based. This city is now the capital of Paraguay, a metropolis complex with more than 500,000 inhabitants with a surrounding region that is heavily altered by anthropogenic impacts. There were indications that hunting pressure in the region remains intense, both for cultural reasons and for obtaining animal protein. Several local actors (e.g., cowhands and indigenous people) have long reported the absence of the red brocket deer in the region. Thus, an alternative location was sought, as close as possible, with preserved forest habitat, evidence of the species’ presence, and no evident geographical barrier concerning the original type location.

The region of the Brazilian city of Foz do Iguaçu, on the Brazil-Paraguay border, 290 km from Asunción, was selected as the target location to collect a specimen. The region comprises the Iguaçu National Park, a continuous block of 185,000 ha of preserved Atlantic Forest, on the banks of the Paraná River, the main river of the Río de La Plata Basin, with direct connection with Paraguay River. An adult female, run over on BR 469, a few meters from Iguaçu National Park, was indicated as a neotype in this study.

#### Animal Samples

The present work had access to a set of individuals (*n* = 53) that allowed a broad analysis of brocket deer species of *Mazama*. The set consisted of samples from *Mazama nana* (*n* = 11); *M. bororo* (*n* = 9); *M. temama* (*n* = 4) and samples of various cytotypes within the *M. americana* complex (*n* = 29). All analyzed specimens are preserved in the Deer Research and Conservation Center (NUPECCE) Museum, Tissue and Cell Banking at São Paulo State University, Jaboticabal city, Brazil. The samples and information came from animals collected directly from nature, injured animals received for treatment, or samples collected from animals in public and private breeding sites with wild origin (Figure 1). The neotypes specimens were present in all datasets (morphology, cytogenetics, molecular), but not all samples were present in all datasets. The information of which sample composed each dataset is detailed in the table presented in SM01.

#### Fecal Samples

Non-invasive genetic sampling was conducted with the collection of fecal samples, which focused on several forest habitats in the central region of South America, more specifically the Brazilian biomes of the Atlantic Forest, Cerrado, Pantanal and southern Amazon region. The collection was carried out in 20 locations and samples from 10 other locations used in previous studies were added (Duarte et al., 2017; Oliveira et al., 2019, 2020) (Figure 1; SM-02). The collection of fecal samples was performed with the support of a detection dog trained to find feces from all deer in the study region. Altogether, 107 days of field work were performed, with an average of 4 h of daily effort. The samples were preserved in absolute ethanol at the ratio of 1 volume of feces to 4 volumes of ethanol (approximately 40 ml) and all had their geographic coordinates noted.

### Cytogenetic Data

#### Sample Preparation and Analysis

For each animal accessed, either alive or recently slaughtered, a skin biopsy of the inguinal region was collected and frozen in liquid nitrogen as described for leucocytes by Duarte et al. (1999) in order to obtain chromosomal preparations from fibroblast culture (Verma and Babu, 1995). Metaphasic chromosomes were subjected to conventional Giemsa staining, C-banding by barium hydroxide solution (Sumner, 1972), Ag-RON silver nitrate staining (Howell and Black, 1980) and G-banding using standard trypsin/Giemsa treatment (Seabright, 1971). We classified the chromosomes according to the ratio of arms as metacentric, submetacentric, or acrocentric (Levan et al., 1969). Relative length (CR) was used to organize chromosome pairs into group A (large two-armed chromosomes, CR ≥ 6%), group C (small two-armed chromosomes, CR < 6%), group D (large one-armed chromosomes, CR ≥ 5%), group E (small one-armed chromosomes, CR < 5%), and group B (B chromosomes, CR < 1.5%) (Cifuentes-Rincón et al., 2020). We assembled the G and C-banding graphical representation with the Adobe Illustrator software.

#### Fluorescent *in situ* Hybridization

Fluorescent *in situ* hybridization (FISH) was performed using bovine whole chromosome painting (WCP) probes on karyotypes of the *M. rufa* and *M. americana* (*sensu stricto*) neotypes, in a male of the Paraná cytotype (T308), and another male of the Carajás cytotype (T166) of the *M. americana* complex (*sensu lato*). Painting probes derived from cattle were selected considering their proven efficiency in karyotypic studies of the family Cervidae (Frohlich et al., 2017; Galindo et al., 2021). Whole chromosomes were isolated by microdissection in the PALM Microlaser system (Carl Zeiss MicroImaging GmbH, Munich, Germany) (Kubickova et al., 2002) or by flow sorting using MoFlo XDP Cell Sorter (Beckman Coulter, Indianapolis, IN, United States) (Frohlich et al., 2017). For amplification and labeling of chromosomal DNA, a DOP-PCR (degenerate oligonucleotide primed polymerase chain reaction) reaction (Telenius et al., 1992) was performed followed by a second PCR with Green-dUTP or Orange-dUTP (Abbott Park, IL, United States) (Kubickova et al., 2002). FISH was performed as presented in Vozdova et al. (2019). Hybridization signals were examined using Zeiss Axio Image Z2 fluorescent microscope (Carl Zeiss Microimaging GmbH, Jena, Germany), equipped with appropriate fluorescent filters, and images were captured using the Metafer Slide Scanning System (MetaSystems, Altlussheim, Germany). Images were analyzed using ISIS3 software (MetaSystems, Altlussheim, Germany).

### Morphological Data

#### Neotype Morphology Description

The animal proposed as a neotype for *Mazama rufa* (Illiger, 1815) was identified as T385 and had its morphology described qualitatively and quantitatively. The specimen was photographed and 14 external body characters (body biometry) were measured using a pendular scale, measuring tape, and common caliper (0.1 mm accuracy) (SM-03). After bone maceration, the skull was photographed at different angles and measured according to the standard of cervid cranial measurements proposed by Rees (1969) and Driesch (1976) with a digital caliper (precision 0.01 mm) (SM-03). The entire skin was removed and treated with a tanning solution to desiccate the material. Aspects of general coat color, chromogenic fields of the body, pigment band patterns in hair from different regions of the body, hair length in different regions of the body, and the occurrence of anteverted hair bands and rounded hair tuft in the tarsal region were examined. The chromogenic fields of the head were also analyzed according to the pattern of Hershkovitz (1982). This qualitative description performed for the *M. rufa* neotype (T385) was compared with the amended description of the *M. americana* neotype (T358) (Cifuentes-Rincón et al., 2020).

#### Morphometric Analyses

External body and skull measurements formed two distinct datasets—body biometry and craniometry. Regarding the 42 animals with morphometric data, 27 (64%) composed the body biometry dataset and 32 (76%) the craniometry dataset, with 17 superimposed in the two datasets (SM-03). To assess the similarity among individuals and a possible discrimination of species and cytotypes, a principal component analysis (PCA) based on the correlation matrix between the variables was performed. The first two eigenvectors with the highest percentage of accumulated variance were used to build the graphs. The size of the confidence ellipses indicates the degree of grouping of the evaluated groups based on a normal distribution considering a coefficient of 0.95. All analyzes were performed using Software R (R Core Team, 2020). In addition, we performed UPGMA cluster analysis with Euclidean distance and 1,000 bootstrap using the “Paleontological Statistics” PAST program (Hammer et al., 2001).

### Molecular Data

#### Tissue and Blood DNA Extraction, Amplification and Sequencing

We followed the phenol-chloroform purification protocol described in Sambrook et al. (1989) for DNA extraction of tissue fragments (skin and spleen) and eventually leukocyte ring. Partial genes, Cytochrome-b (Cytb; 978 bp), NADH dehydrogenase subunit 5 (ND5; 1128 bp), and control region (Dloop; 454 bp) were amplified and sequenced using primers described in the literature that can be found together with detailed protocols in SM-04. All amplicons were purified using the Wizard^®^ SV gel and PCR Clean-Up System kit (Promega) and sequenced in both directions in an ABI 3730XL automated sequencer. The forward and reverse sequences were exported to the BioEdit 7.2.6 program (Hall, 1999) and had their electropherograms visually reviewed for quality control and assembly of a consensus sequence of each sample. All sequences produced from tissue samples in this study were deposited in Genbank with accession number MZ488858 to MZ488910 (SM01).

#### Molecular Alignment, Composition, and Model Selection

The tissue dataset was composed by sequences from 38 animals from this study and additional sequences obtained from mitogenomes deposited in GenBank belonging to *M. americana* and *Odocoileus virginianus*. The species *Rangifer tarandus*, *Alces alces* and *Capreolus pygargus* were also obtained from GenBank and were used as outgroup in the phylogenetic analyses (SM01). The consensus sequences of each amplicon and the sequences obtained from GenBank were aligned on the Mafft online server (Katoh et al., 2018), reviewed and trimmed using the Bioedit 7.2.6 software. The alignments from each region were then concatenated using the Mesquite 3.61 (Maddison and Maddison, 2019) resulting in a final alignment of 2560 bp. Polymorphism and saturation were characterized using MEGA X and tests by Xia et al. (2003) using the DAMBE 7.2 software (Xia, 2018). These are presented in SM-04. The selection of the best evolutionary model was determined by data partition analysis with the Partition Finder 2 software on the CIPRES Science Gateway online platform (Miller et al., 2011). Data partitions were tested by mtDNA region and by the Cytb and ND5 codon position. The best scheme was selected among all possible combinations through the smallest Bayesian Inference Criterion (BIC) value. The selection of the best model for each subset of the scheme was also selected through the lowest BIC value among 40 possible evolutionary models in order to be applied into the BEAST package (SM-04).

#### Molecular Phylogenetic and Species Delimitation Analysis

The phylogenetic analysis was performed using the Bayesian Inference (BI) criterion in the BEAST 1.10.4 software package, running parameters implemented through BEAUti, and the analysis performed through the CIPRES Science Gateway online server. The Monte Carlo Markov Chains analysis included 10 million generations, sampled every 1,000 generations performed in three independent runs. Data were combined using the Log Combiner app, the best tree were summarized with a 30% burn-in, and the values of posterior probability (PP) node support were accessed with Tree Annotator. We verified the convergence of the analysis with Tracer v.1.7, considering satisfactory when it presented ESS (Estimated Sample Size) values greater than 200.

We used the General Mixed Yule Coalescent (GMYC) method to identify molecular operational taxonomic units (MOTUs) through the SPLITS software package (Monaghan et al., 2009) in the R program. For this analysis, we used the phylogenetic hypothesis represented by the ultrametric BI-tree considering the ingroup. The GMYC model hypothesis was tested towards single and multiple thresholds. The hypothesis that the GMYC and null models were different was not rejected when presenting *p* < 0.05.

### Distribution Data

#### Fecal DNA Analysis

Genetic species identification was necessary due to the presence of sympatric deer species in the fecal sampling sites (*Mazama gouazoubira, Mazama nemorivaga, Ozotoceros bezoarticus*) in which fecal morphological identification is not a valid option (Costa et al., 2017). Furthermore, the differentiation between the *M. americana* complex and *M. rufa* was never tested and should also rely on genetic identification. Fecal DNA was extracted using the QIAmp Fast DNA Stool MiniKit (QIAGEN) kit and all lab procedures were made in an exclusive forensic DNA room encompassing blank controls for contamination detection. Species identification was conducted with a multiple small mtDNA fragment (∼250 bp) amplification and sequencing strategy that included five regions, internal to the previously sequenced amplicons from the tissue dataset. The protocol proposed by González et al. (2009) was used to sort out *M. americana* complex samples from the other species. After that, we selected 2–3 *M. americana* complex fecal samples per site to conduct *M. rufa* identification. In this step, the complete five mtDNA regions were sequenced and the final alignment (1103 bp) was analyzed in a BI phylogeny using our tissue dataset as reference sequences. All sequences produced from fecal samples in this study were deposited in Genbank with accession number MZ521085 to MZ521234 (SM04).

#### Distribution Modeling

In order to generate distribution models, we used all fecal samples from sites where *M. rufa* was identified as the red brocket present to compose our occurrence records (*n* = 49; SM-02). We used six previously interpolated environmental variables at 30 arc-seconds (approx. 1 km) resolution and clipped them to our modeling scope. Our modeling scope consisted of a 1000 km buffer around our occurrence records and constrained by IUCN *M. americana* geographical distribution plus a 50 km buffer. The variables we selected are recognized to be highly influential in neotropical forest deer distributions (Duarte et al., 2017; Oliveira et al., 2019, 2020), these were: percentage of tree cover (Hansen et al., 2003), altitude (Valeriano, 2008), slope, temperature seasonality (BIO4), annual precipitation (BIO12) and precipitation of the driest month (BIO14) (Hijmans et al., 2005). To ensure these variables are not correlated in our modeling scope we performed a Pearson’s correlation test to confirm *r* < 0.8.

We performed the analysis using Maxent software version 3.4.1 (Phillips and Schapire, 2004; Phillips et al., 2006). The model was “trained” using 70% of the occurrence records and tested with the remaining 30% of the records (Pearson, 2010). The occurrence records were sampled using the bootstrap method, with ten random partitions and substitutions. We assessed each species average model using the area under the curve (AUC), calculation of the omission error, and binomial test for comparison of two proportions (Fielding and Bell, 1997; Elith et al., 2006; Phillips and Dudík, 2008).

## Results

### Neotype Designation and Species Diagnosis

#### Amended description of *Mazama rufa* (Illiger, 1815) (MAMMALIA, CERVIDAE):

**FIGURE 2:**
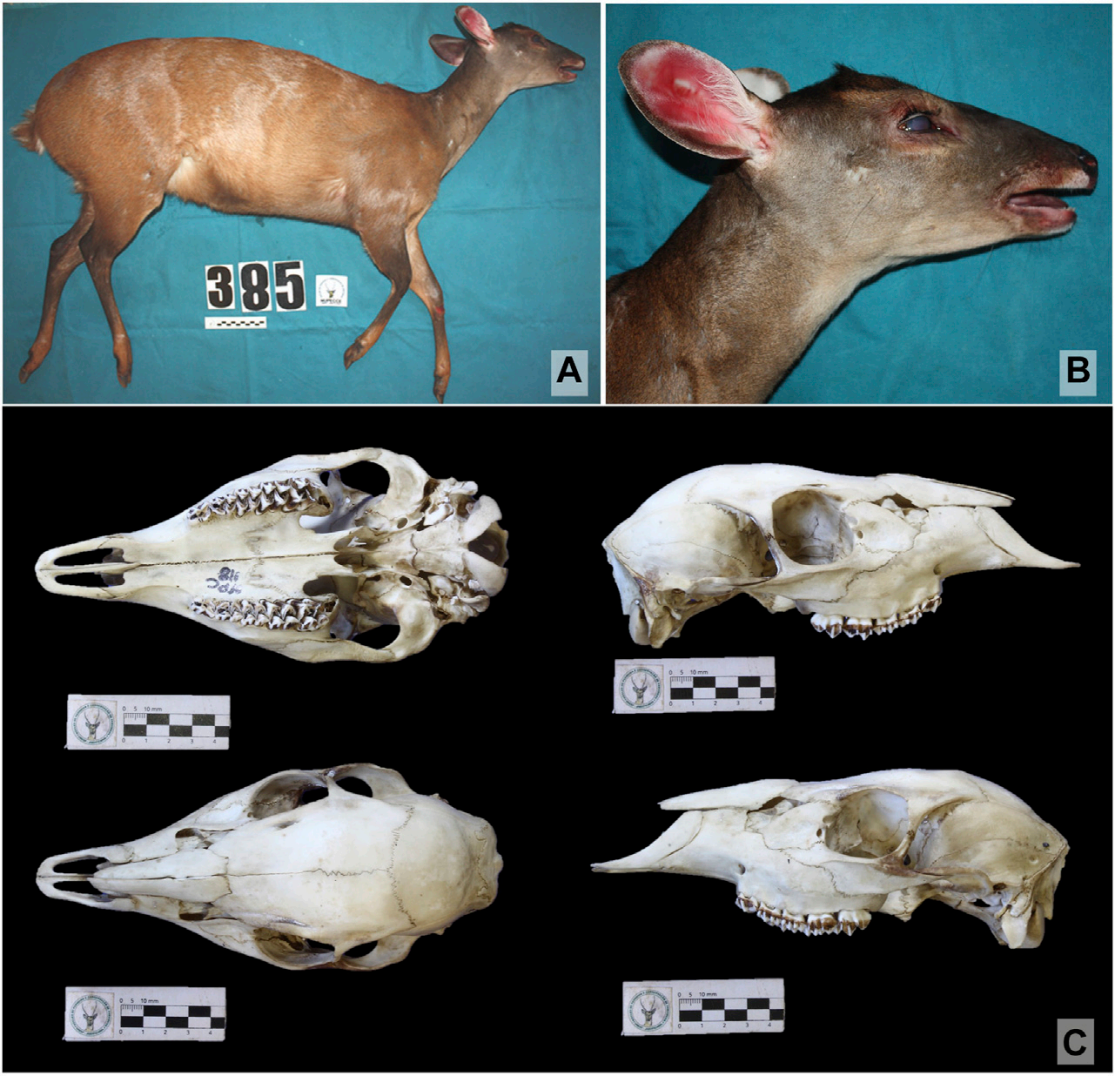
Images of the female specimen designated as the *Mazama rufa* neotype (T385) (Illiger, 1815) (Artiodactyla: Cervidae). **(A)** lateral view of the body; **(B)** detailed view of the head; **(C)** dorsal, ventral, left lateral, and right lateral view of the skull. Material deposited at the NUPECCE museum identified under voucher NPC118.

Predominantly reddish-brown at laterals, with blackened areas in the head region, neck, thorax, distal regions of hind, and forelimbs. Lumbar line, darker than the predominant bright reddish coloration. Posterior region of the hips and tail dorsum with reddish-brown color. Whitish Inguinal region whitish as well as its inner thigh. Presence of a tarsal hair tuft. Whitish tail in ventral region, while reddish in dorsal region. Color of the lateral area of limbs well-defined, bright reddish in the proximal area and dark brown in the distal part, as well in the dorsal line and rostral area. White inner thigh and inguinal region. Proximal region of hind limbs reddish brown medially. Presence of tarsal hair tuft. Longer hair in the basal region of the ear. Anteverted hair band at the nape of the neck. Sides of the head with brown coloration. Small, inclined ears, white auricular border and blackened posterior auricular border. Whitish anterobasal auricular patch and brown reddish posterobasal auricular patch. Outer auricular surface blackish brown. Yellowish-red upper orbital band. Inferior orbital band with the same rostral coloration, over a yellow band. Absence of superciliary spot. Nasal patch present, followed by a dark lateral rostral band and pale lateral rostral band. Mental white patch. Reddish brown mandibular band. Buccal patch present. Presence of white gular patch. Light brown neck at the ventral region. Skull with shallow lacrimal fossa not deepened, two lacrimal foramina internally to the edge of the orbit, separated from each other. Vomerine septum typical of Capreolinae. Small tympanic bulla. Short inclined pedicles and rectangular pre-orbital region (Figure 2).

*Synonymy*: *Cervus gouazoupita* G. Fischer, 1814:465*.* Type locality Paraguay; based on Azara (1801) *Troisième cerf ou Gouazoupita*; *Mazama pita* Rafinesque, 1817:363. Type locality Paraguay; based on Azara (1801) *Troisième cerf ou Gouazoupita*.

*Amended diagnosis.*—The amended diagnosis of *Mazama rufa* adds to the original description (Azara, 1801) other approaches different from morphology. Chromosomic diploid number ranging from 52 to 53, fundamental number of 56, one pair of submetacentric autosomes, 48 acrocentric autosomes and multiple sexual system XY1Y2 due to an X-autosomal fusion. Thus, differing from *M. americana* (Erxleben, 1777), type species of the genus *Mazama* that showed chromosomic diploid number of 45 and fundamental number 51.

*Neotype* (*T385*)*. —* Avenida das Cataratas, número 2,264. BR 469. Foz de Iguaçu, Paraná, Brazil (25º36′22″S, 54º29′54″W; datum WGS84). Deposit number: NPC118; full skull, post skull, taxidermied skin; live cells and tissues. Karyotype: 2n = 52, FN = 56 (female). Mithocondrial DNA sequences deposited in GenBank under accession numbers: MZ488852; MZ488925; MZ488894. Specimen deposited in Museum, Tissue and Cell Bank of the Deer Research and Conservation Center (NUPECCE) of the Faculty of Agricultural and Veterinary Sciences of the São Paulo State University (UNESP), Jaboticabal campus, Brazil.

### Cytogenetic Data

**FIGURE 3:**
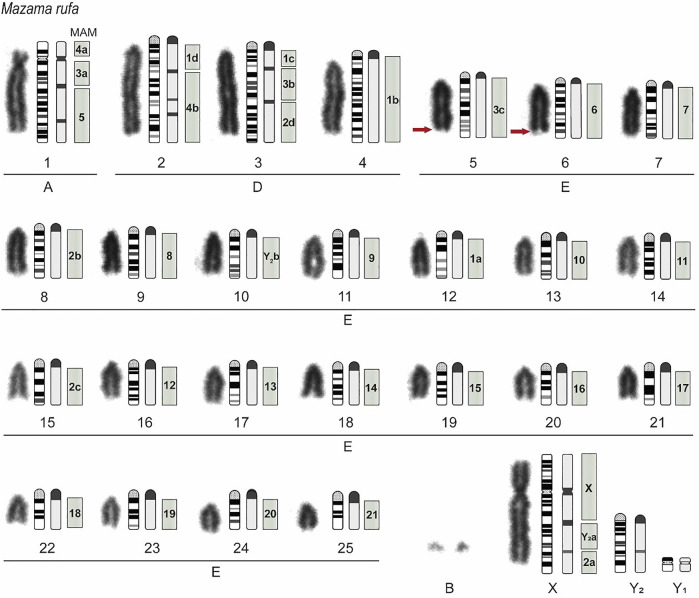
*Mazama rufa* neotype karyotype (2n = 52 + 2–6 B, FN = 56). Conventional Giemsa staining, schematic representation of G and C banding, and homologies to *Mazama americana* (MAM) chromosomes are displayed in a left-to-right direction. The gray squares indicate the homologies with MAM chromosomes. The letters a, b, c, and d represent regions of the chromosome that were homologous to *M. rufa*. For the submetacentric pairs MAM1 and MAM2: a = p arm, b = proximal q arm, c = medial q arm, and d = terminal q arm. For acrocentric pair MAM3: a = proximal region, b = medial region, and c = terminal region. MAM4 and MAMY2: a = proximal and b = terminal region. The red arrows indicate the position of the nucleolus organizer regions.

The *M. rufa* neotype presented a karyotype constitution of 2n = 52 and FN = 56. The chromosome measurement by relative length classified pair 1 belonging to Group A, pairs 2 to 4 to Group D, pairs 5 to 25 to Group E. The X chromosome was submetacentric (Figure 3). Two to six supernumerary chromosomes (Bs) were observed at metaphases. This autosomal pattern observed for the neotype corresponds to the Paraná cytotype of *M. americana* (Abril et al., 2010), which from now on corresponds to the species *M. rufa*. Thus, the description of the sex chromosomes was also performed for a male specimen (T308). The sexual system was XY1Y2 due to an X-autosomal fusion. The male presented a karyotypic constitution of 2n = 53, FN = 56, Y1 was the smallest chromosome, and Y2 a medium acrocentric.

C-banding showed constitutive heterochromatin blocks in the centromeric region of all autosomal chromosomes, two interstitial bands in the q arm of pairs 1 and 3, and three discrete interstitial bands in pair 2. The sex chromosome X had a strong interstitial band in the proximal region of the q arm and a discrete interstitial band in the distal region of the same arm. Chromosome Y_1_ is completely euchromatic and chromosome Y_2_ has a discrete interstitial band in the medial region of its arm. The B chromosomes showed variation in their heterochromatin content. While some B were completely heterochromatic, others had only interstitials heterochromatin bands. The nucleolus organizer regions were localized in the distal region of the long arms of both chromosomes from pairs 5 and 6. Figure 3 shows the graphical representation of the G and C-banded *M. rufa* chromosomes, which is of considerable importance in comparative cytogenetics.

**FIGURE 4:**
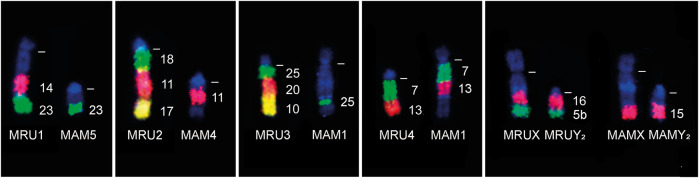
FISH results demonstrating some of chromosomal rearrangements differences between the species *Mazama rufa* (MRU) and *Mazama americana* (MAM) using cattle chromosome painting probes (indicated on the right). The dashed white lines indicate the position of centromeres.

The comparative cytogenetics analysis with G-band and cattle (BTA) WCP probes (Figure 4) showed that *M. rufa* (MRU) and *M. americana* (*sensu stricto*) (MAM, 2n = 45, FN = 51) accumulated different rearrangements during their karyotype evolution. The p arm of the submetacentric MRU1 is homologous to the proximal region of MAM4 (Figure 3, 4a), and the q arm to the proximal region of MAM3 (Figure 3, 3a) and to the MAM5 chromosome. The large acrocentric MRU2 showed homologies with the terminal region of MAM1 (Figure 3, 1d) and the distal region of MAM4 (Figure 3, 4b). The MRU3 pair corresponded to the distal regions of MAM1 (Figure 3, 1c) and MAM2 (Figure 3, 2d) and to the proximal region of MAM3 (Figure 3, 3b). MRU4 and MRU12 are homologous to the proximal region of the MAM1 q arm (Figure 3, 1b) and to the p arm (Figure 3, 1a), respectively. MRU5 corresponded to the distal region of MAM3 (Figure 3, 3c). MRU8 and MRU15 is homologous to the proximal region of the q arm of MAM2 (Figure 3, 2b and 2c). Finally, acrocentric pairs MRU 6, 7, 9, 11, 13, 14 and MRU 16 to 25 were homologous to one MAM acrocentric chromosome.

The composition of sex chromosomes also differed between the two species. Both species have a multiple sex chromosome system XY_1_Y_2_ formed by X-autosomal fusions during the divergence from a common ancestor. However, the distal parts of X and Y_2_ were not homologous between the two species. In *M. rufa*, the distal regions of X and Y_2_ correspond to the p arm of MAM2 (Figures 3, 2a), while the distal part of X and Y_2_ of *M. americana* corresponds to MRU10. The Y_2_ chromosomes of these species were partially homologous, demonstrating that the first X-autosomal fusion probably occurred in an ancestral karyotype of the group and the second fusion was formed later in each species (Figure 3). Thus, at least 15 rearrangements separate *M. rufa* and *M. americana* karyotypes.

Furthermore, the *M. americana* Carajás cytotype (2n = 50, FN = 54) showed the fixation of a tandem fusion between two acrocentric chromosomes of *Mazama rufa*. This fusion occurred between MRU5 and MRU8, forming CA3, while the other chromosomes corresponded between these species. The location of the hybridization signals for each bovine probe used is specified in SM-05.

### Morphological Data

In the body biometric dataset, none of the variables presented extreme outliers (>3SD in relation to the mean) and the total proportion of lost data was 5.16%. In the craniometry dataset, only one variable for a single animal showed extreme outlier and was removed, and the total proportion of lost data was 4.08%. Descriptive analyses (mean, standard deviation, maximum and maximum) of both datasets are presented in SM-03.

**FIGURE 5:**
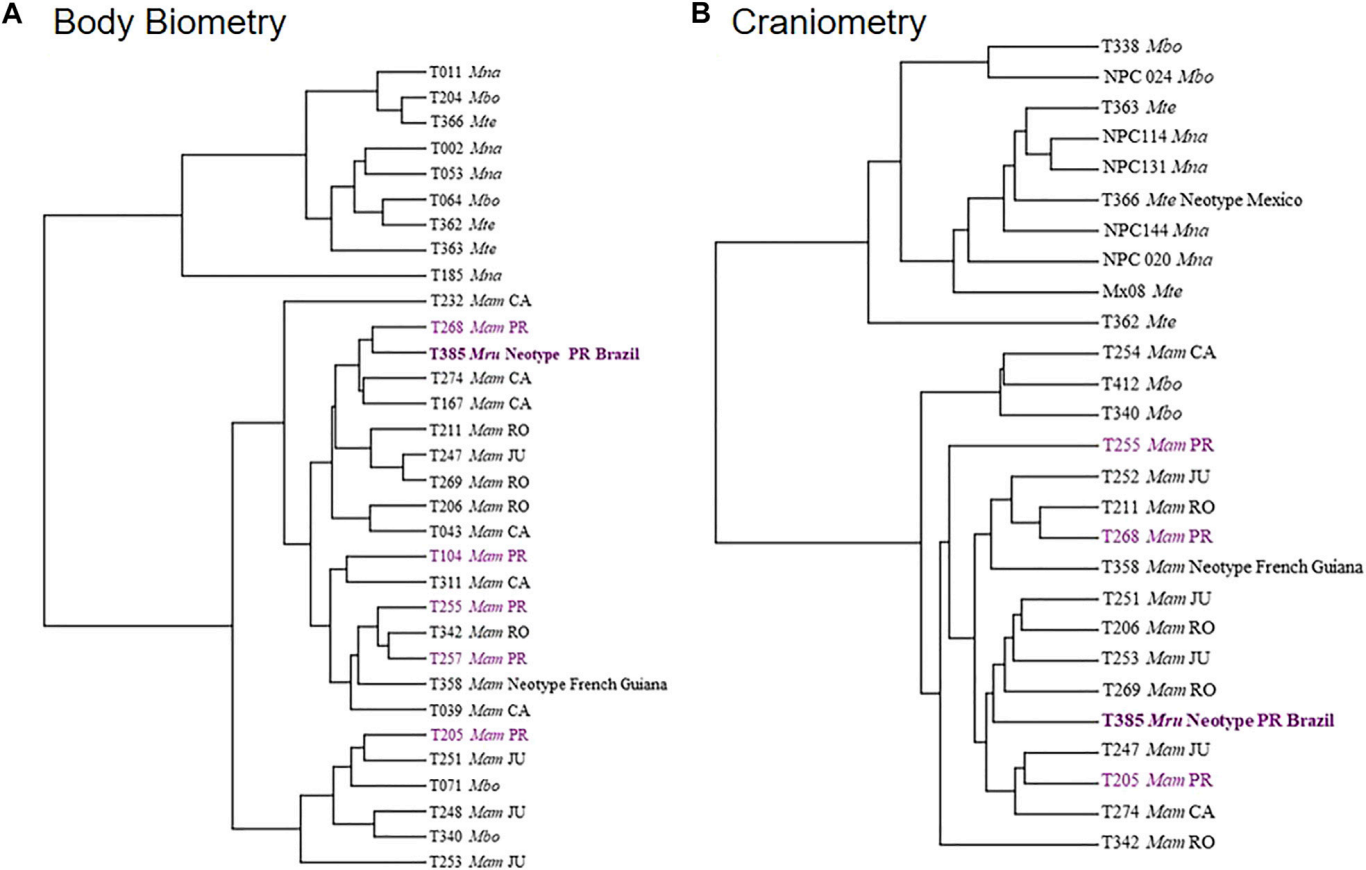
Distance tree made with body biometrics **(A)** and craniometry **(B)** datasets using the UPGMA method for the species of the genus *Mazama* (*Mna*–*M. nana*; *Mbo*–*M. bororo*; *Mte*–*M. temama*; *Mam*–*M. americana*) and different *Mazama americana* cytotypes (RO–Rondônia; JU–Juína; CA—Carajás; PR—Paraná).

**FIGURE 6:**
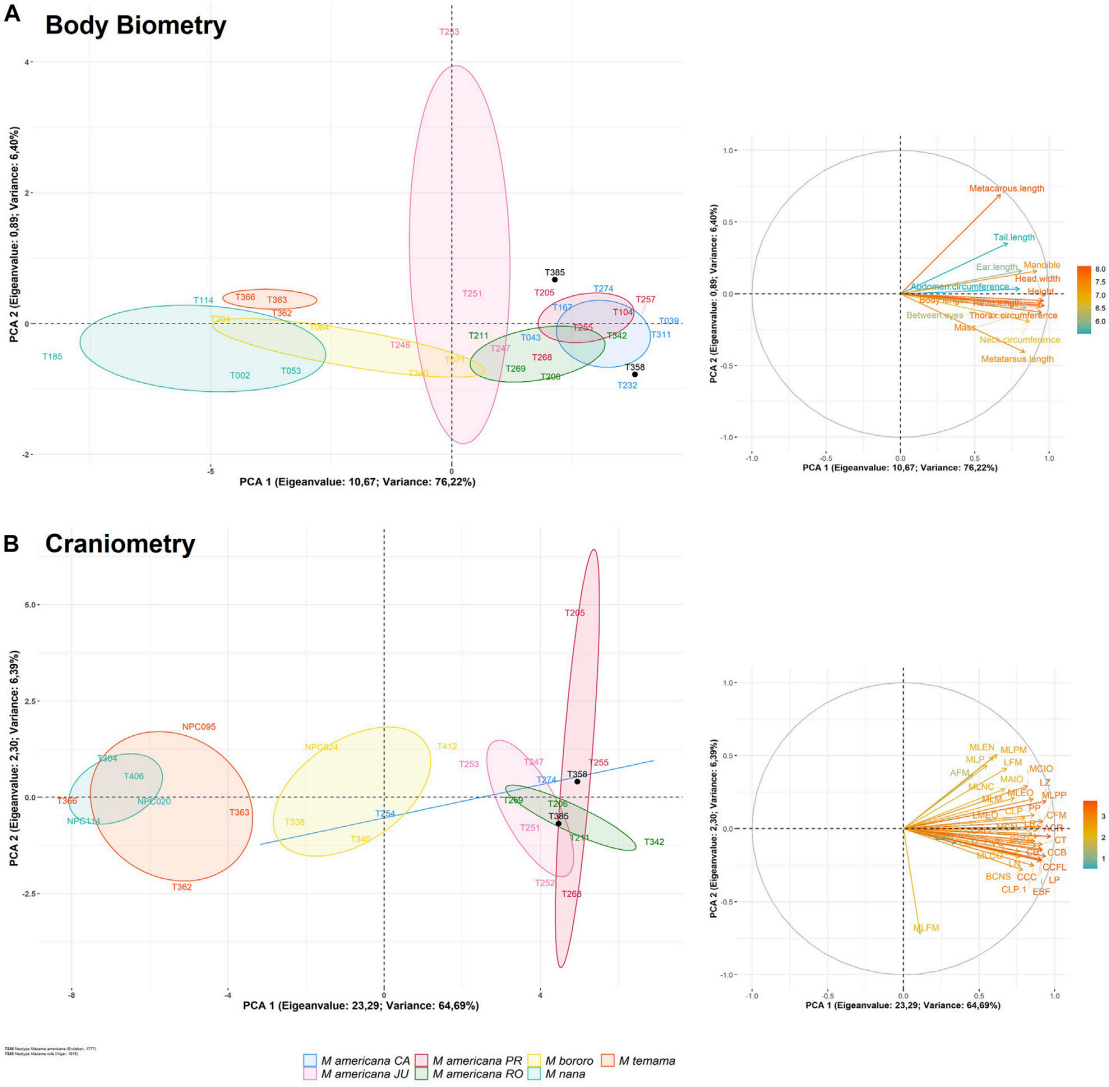
Morphology PCA results of body biometry **(A)** and craniometry **(B)** datasets represented by scatterplot of first and second principal components with 95% confidence ellipses clustered by the test groups **(left)** and loading plot of the environmental parameters **(right)**. The colored scale bar represents the correlation of each parameter to the first principal component.

The UPGMA distance tree from the two morphometry datasets subdivided the specimens into two groups (Figure 5). The first group corresponds to the *M. americana* complex (*sensu lato*), including the neotype from French Guiana, the neotype proposed for *M. rufa* and specimens from Brazilian cytotypes. The second group was represented by the *M. nana* and *M. temama*. Finaly, *M. bororo* was subdivided with two individuals in each group. All specimens were positioned in a mixed manner within each main group where neither species nor cytotypes were grouped together (Figure 5). In the PCA analysis, the two datasets presented similar results to those of the UPGMA with a high overlap among species and among cytotypes, but some distinctions were observed in the craniometry dataset (Figure 6). It was possible to discriminate *M. bororo* from the other animals, characterizing the species as an intermediate morphological taxon between the *M. americana* complex, *M. nana* and *M. temama*. The *M. americana* complex, represented by the neotypes and by the Paraná (*M. rufa*), Carajás, Rondônia, and Juína cytotypes, presented the greatest overlap, reinforcing their cryptic complex aspect. In both datasets (body and skull) it is noteworthy that despite the large number of variables, all showed a high association with PCA1, which was able to concentrate respectively 76 and 64% of the data variation in each dataset.

The qualitative characteristics of coat color and hair length showed subtle differences between the amended description of the *M. rufa* (Illiger 1815) neotype and the *M. americana* (Cifuentes-Rincón et al., 2020) neotype. The *M. rufa* species presented a reddish-brown color pattern in the anterobasal region of the ears and in the inner proximal region of the hind limbs, with the presence of long hairs in the basal ears regions and medial to hind limbs. This contrasts with the *M. americana* neotype, which showed a whiter pattern in each of these areas, and an absence of hair at the base of the ears, and the inner proximal region of both limbs (Cifuentes-Rincón et al., 2020).

### Molecular Data

**FIGURE 7:**
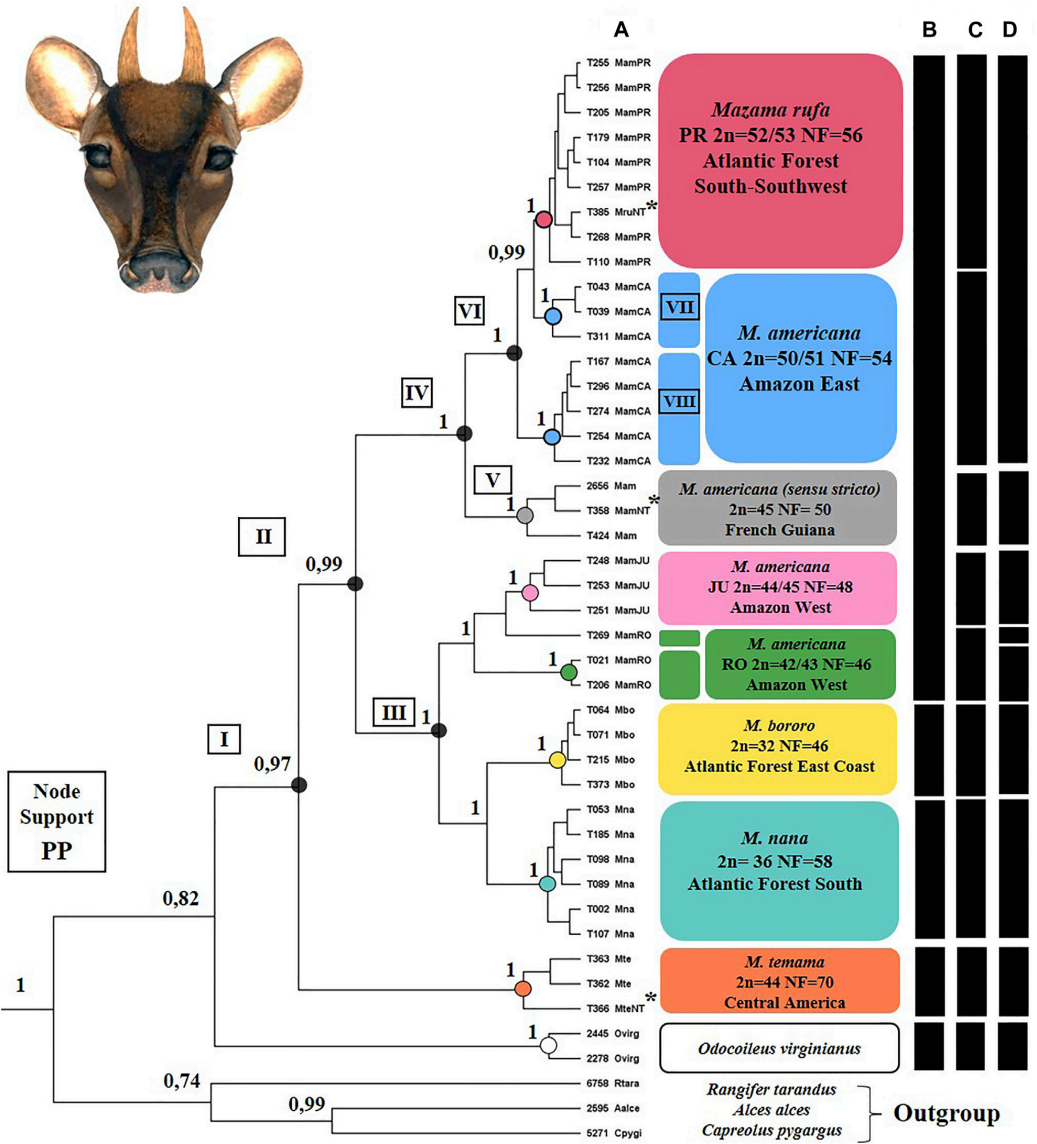
Summary of the phylogenetic inference results and molecular species delimitation for the *Mazama americana* complex (Artiodactyla: Cervidae). **(A)** Ultrametric tree representing the phylogenetic hypothesis generated by Bayesian Inference by the BEAST package. The support values of the clades are represented by the posterior probability (PP 0-1). Numbers I to VII identify clades highlighted in the text. **(B)** Present taxonomic hypothesis. **(C)** Cytotype taxonomic hypothesis. **(D)** Molecular taxonomic hypothesis represented by the Molecular Operational Taxonomic Units obtained by the single-threshold GMYC coalescence method (p = 0.0018). Sequences identified with T### represent sampled animals and sequences identified by #### are those obtained from GenBank, * indicates neotypes.

The phylogenetic hypothesis obtained by BI in the tissue dataset presented most clades with good support (PP > 0.95 Figure 7A). Molecular species delimitation analysis showed the GMYC model significantly higher than the null model for the single threshold (p = 0.0018), while the GMYC model was not significantly higher for multiple thresholds (p = 0.0959) (SM-04). Thus, the single threshold model identified nine MOTUs (Figure 7D) that were compared with delimitations based on the current taxonomy of the analyzed species (Merino and Rossi, 2010; Figure 7B) and on the cytotypes taxonomic hypothesis (Abril et al., 2010; Cursino et al., 2014; Salviano et al., 2017; Galindo et al., 2021; Figure 7C).

The phylogenetic hypothesis recovered the sampled *Mazama* species as a well-supported monophyletic group (clade I) in relation to genus *Odocoileus* (Figure 7). *Mazama temama* was positioned as a basal species in the group and was recovered as a monophyletic clade and single MOTU. The rest of the genus (clade II) was divided into two main clades, one with *M. americana* Juína and Rondônia cytotypes, *M. nana,* and *M. bororo* (III), and another with only *M. americana* (*sensu lato*) lineages (IV). In clade III, we observed a reciprocal monophyly relationship between *M. nana* and *M. bororo*, and each one was indicated as a distinct MOTU. Also in clade III, a sub-clade composed of the Juína and Rondônia *M. americana* (*sensu lato*) cytotypes was positioned as a sister group to *M. nana*-*M. bororo*. Those did not show reciprocal monophyly because one Rondônia sample (T269) was grouped in the Juína clade. However, the GMYC analysis indicated the clades of each cytotype as two distinct MOTUS and recognize this sample as belonging to a third potential species. In the other main clade (IV), only with *M. americana* (*sensu lato*) samples, two MOTUs were delimited, each one with a distinct neotype. The *M. americana* neotype (T358) was grouped with another sample from French Guiana and with animal T424 from the extreme north of Brazil, forming an exclusive clade (V) that represents *M. americana* (*sensu stricto*). This clade, in turn, was the sister group of the *M. rufa* neotype (T385) clade (VI), whose MOTU was composed of the Carajás and Paraná cytotypes. The animals of the Paraná cytotype were recovered in a well-supported monophyletic clade, but nested to the two clades composed by the Carajás cytotype animals (VII and VIII), with no reciprocal monophyletic relationship observed among the two cytotypes.

### Distribution Data

Non-invasive sampling resulted in 241 fecal samples that, coupled with the previously red brocket samples collected, resulted in 15 sites with *M. rufa* confirmed presence (Figure 1; SM-02). Our distribution model had a high AUC value (AUC = 0.908, SD 0.018), low omission errors (0.05) for a fixed cumulative value 10 Logistic threshold and were significant (*p* < 0.05) for the binomial test. It indicated a potential distribution of *M. rufa* across southeast Brazil extending to eastern Paraguay and Argentina, northward to eastern Bolivia and southern Amazon (Figure 1). The high suitability core areas were close to the neotype capture site encompassing forests fragments in the Misiones province in Argentina, and other fragmented areas in the border between Paraguay and Bolivia. This analysis also showed the habitat suitability connection between the neotype locality and the original type locality described by Illiger (1815) in the Asunción city region.

## Discussion

### Cytogenetics

The difference between *M. americana* (*sensu stricto*) and *M. rufa* (former Paraná cytotype) was suggested to envolve two tandem fusions, two Robertsonian translocations and a pericentric inversion based on the hypothetical *M. americana* ancestral karyotype (Abril et al., 2010; Cifuentes-Rincón et al., 2020). However, the association of the G-Band with cattle WCP probes demonstrated that *M. americana* (*sensu stricto*) (2n = 45, FN = 51) and *M. rufa* (2n = 52/53, FN = 56) underwent surprisingly distinct chromosomal rearrangements during their karyotypic evolutions and diverge in at least 15 rearrangements. These findings support the hypothesis that *M. rufa* is a distinct species from *M. americana*, given that smaller karyotypic differences among other *M. americana* cytotypes resulted in complete sterility of their hybrids (Cursino et al., 2014; Salviano et al., 2017). Accumulation of chromosomal rearrangements leads to errors in meiosis and chromosome pairing, recombination suppression, errors in meiotic segregation, and subsequent germ cell or zygote death (Rieseberg, 2001; Villagómez and Pinton, 2008; Dobigny et al., 2017).

On the other hand, the difference of one tandem fusion between *M. rufa* and the Carajás cytotype (MRU 5/8, forming CA3) has been described (Abril et al., 2010; Galindo et al., 2021). The potential hybridization between both populations would lead to the formation of individuals carrying one tandem fusion in heterozygosis, considering the conservative observation of absence of a prezygotic barrier in captivity (Carranza et al., 2018). Such rearrangement is considered deleterious and with a hypothetical 50% reduction in the production of balanced gamete (King, 1993), resulting in reduced fertility of the carrier (Moritz, 1986; Pillay et al., 1995; Long, 1996; Galindo et al., 2021). Nevertheless, spermiogram and testicular histology analysis failed to demonstrate a significant reduction in the reproductive fitness of hybrids carrying one tandem fusion in heterozygosis when compared to purebred animal. These were classified as sub-fertile, which would reduce the possibility of establishing a post-zygotic reproductive barrier between populations (Salviano et al., 2017). In this context, a recent study evaluated the meiotic segregation of hybrids with one tandem fusion in heterozygosis in the *M. americana* complex, including a hybrid between the Carajás and Paraná (*M. rufa*) cytotypes, obtaining a rate of ∼30% gametic unbalance (Galindo et al., 2021). Considering the chance of successful reproduction in a backcross breeding with animals from the parent populations (∼35% with the presence or ∼35% with the absence of tandem fusion in balanced gametes) this reduction in hybrid fertility might be even greater (∼65% of gametic unbalance). Thus, the difference of one tandem fusion among populations is suggested as an efficient post-zygotic reproductive barrier, similar to that observed for *Otomys irroratus* (Pillay et al., 1995). Furthermore, it is important to indicate the apparent ongoing process of karyotypic evolution of the Carajás cytotype, demonstrated by the presence of chromosomal polymorphisms such as centromeric fusion in heterozygosis and homozygosis (Galindo et al., 2021). The possibility of crossing these latter cytogenetic variants with *M. rufa* could result in the production of hybrids carrying tandem fusion and centromeric fusion, both in heterozygosis. This interaction among different chromosomal rearrangements could result in increased rates of unbalanced gametes, as already observed in a hybrid of the *M. americana* complex carrying both rearrangements (43.05% of sperm with aneuploidy), with a subsequent gametic unbalance of ∼70%, regarding any parent population (Galindo et al., 2021). This evidence supports the hypothesis of considering *M. rufa* and the animals of the Carajás cytotype as distinct species.

### Morphology

The external morphological similarity (body biometry) among *M. americana* lineages and other *Mazama* species has been previously demonstrated (Duarte et al., 2008). A similar result was also obtained in a craniometry analysis of *M. americana* lineages, but the comparison with other species of the genus was limited (Cifuentes-Rincón et al., 2020). Our results showed a level of distinction among different species in the body biometric data, specifically between *M. nana*, *M. temama* and the other evaluated species. In the craniometry data, *M. bororo* was isolated, with only one specimen of *M. americana* overlapping it. Nevertheless, this distinction of *M. bororo* was not statistically supported in Rossi (2000) assessment, nor is it confirmed in the broad overlap of forest deer craniometry reported by Cassini and Toledo (2021).

The positioning of the *M. americana* neotype was close to the *M. rufa* neotype and quite overlapping with other *M. rufa* specimens (Paraná cytotype) and also Carajás cytotype specimens in both datasets. Qualitative analysis, however, identified coat features that appreciably distinguish the neotypes of the two species. This represents a first step towards finding diagnostic morphological characters, which must be tested within a broader sample. The detailed analysis of deposited specimens using a *post hoc* approach to compare the *M. americana* genetic lineages may contribute to the identification of diagnostic characters. This type of analysis is also called “reverse taxonomy” (Markmann and Tautz, 2005) and can be an interesting way to identify morphological differences, reinforce the delimitations observed by genetic data and organize scientific collections where a high primary identification error in their *Mazama* vouchers have been observed (Michaloudi et al., 2018; Mantellatto et al., 2020).

### Mitochondrial DNA Phylogeny

Mitochondrial genes have been systematically used in several works that aimed to recover the phylogenetic relationships among species of the tribe Odocoileini in the last 2 decades (Gilbert et al., 2006; Duarte et al., 2008; Gutiérrez et al., 2015, 2017; Escobedo-Morales et al., 2016; Heckeberg et al., 2016; Cifuentes-Rincón et al., 2020; Heckeberg, 2020). Some studies tested the use of nuclear regions (a-lactalbumin; protein kinase C iota; satellite DNA) but they were not informative in recovering phylogenetic relationships between recently divergent species (Gilbert et al., 2006; Heckeberg, 2020; Vozdova et al., 2021). Although the present work brought the widest sampling (number of vouchers and mitochondrial sequence size) in the *M. americana* complex, it still represents a single-locus analysis that limits interpretation of the results, given that mtDNA gene-tree may underestimate introgression and hybridization processes (Shaw, 2002). Given that results can diverge depending on the analyzed regions, a multi-loci analysis involving nuclear markers and species-trees would be a more precise approach to recover phylogenetic relationships, divergence times, and MOTU delimitation in an unbiased analysis (Igea et al., 2015; Dool et al., 2016).

The polyphyletic status of *Mazama americana* was revealed when the species was recovered into two clades that formed a polytomy with *Odocoileus virginianus* and were nested with *M. nana* and *M. bororo* (Duarte et al., 2008). Different studies observed the same results in phylogenetic analyses involving more species from the tribe Odocoileini (Escobedo-Morales et al., 2016; Heckeberg et al., 2016; Gutiérrez et al., 2017; Heckeberg, 2020). The problem is that studies restricted to Cytb gene did not present sufficient resolution, and recovered the specimens in a mixed form, without clades consistent with the region of origin, chromosomal lineage, or cytotype (Duarte et al., 2008; Gutiérrez et al., 2017). On the other hand, more informative alignments recovered each chromosomal lineage in distinct and well-supported clades (Abril et al., 2010). In these analyses, it was possible to observe the sister-group relationship between the *M. americana* lower diploid number lineage and *M. bororo*, and between the higher diploid number lineage and *M. americana* (*sensu stricto*) (Cifuentes-Rincón et al., 2020). Nevertheless, previous works have not actually tested reciprocal monophyly between closely related cytotypes, such as Rondônia x Juina (lower 2n) or Paraná x Carajás (higher 2n). Our results were consistent with previously phylogeny regarding chromosomal lineages and species, and for the first time tested the relationship between cytotypes of close divergence. The absence of reciprocal monophyly between both pairs of close cytotypes would invalidate the hypothesis that they represent distinct species, taking the strict phylogenetic species concept into account (Cracraft, 1983). However, this result should be evaluated with caution as it may be the result of insufficient polymorphism in the analyzed sequence or the bias from incomplete lineage sorting in an analysis restricted to a single locus.

### Revalidation and Delimitation of *Mazama rufa* (Illiger 1815)

Modern taxonomy proposes an integrative method in which different data sets could result in a greater accumulation of evidence to support taxonomic revisions (Padial et al., 2010; Fujita et al., 2012). More interesting than searching for consistency in the different sources of evidence it is necessary to search for the information that is biological meaningful for the taxon in question (Padial et al., 2010). The definition of a taxonomic hypothesis in view of contrasting data can be equally relevant if well-grounded in the evolutionary processes that drive the speciation of the analyzed group (Fišer et al., 2018).

The incongruity between morphological and genetic data should not represent a problem given that morphological similarity in cryptic species is understood as a result of three possible evolutionary mechanisms: recent divergence, niche phylogenetic conservatism, or morphological convergence (Fišer et al., 2018). The niche phylogenetic conservatism hypothesis is interesting for the *M. americana* complex as it considers specialist species under strong selective pressure (Fišer et al., 2018). In this regard, brocket deer species are considered forest specialists (Weber and Gonzalez, 2003; Duarte et al., 2017; Oliveira et al., 2019; González and Duarte, 2020) and the selective pressure of this environment has already been identified as a determinant feature in the morphological convergence of different lineages of the genus *Mazama* and *Pudu* (Gilbert et al., 2006; Duarte et al., 2008; Heckeberg, 2020). In these taxa, for example, spiked antlers are understood as characters that emerged independently throughout the evolution of neotropical deer (Gilbert et al., 2006; Duarte et al., 2008; Heckeberg, 2020). In the case of the red brocket lineage (*M. temama*, *M. nana*, *M. bororo*, *M. rufa* and *M. americana* complex), a group of close-sister species, it would not be the case of convergence, but of morphological stasis resulting from the maintenance of the niche under similar environmental pressure of forests habitats. Thus, the absence of morphological differentiation does not necessarily represent persistence of gene flow among *M. americana* cytotypes and therefore does not invalidate the recognition of distict species such as the present description of *M. rufa*.

Molecular data and cytogenetic data converged in indicating *Mazama rufa* (Illiger, 1815) as a distinct species in relation to *Mazama americana* (Erxleben, 1777). The role of chromosomal divergence is related to the gene flow reduction between divergent populations of mammals (Rieseberg, 2001), which can lead to a process of isolation and speciation as already discussed above. The operational biological species concept is the core of this hypothesis, in which reproductive isolation would unequivocally identify the existence of different species (Mayr, 1942). It is clear that both species represent independent evolutionary lineages within the ontological concept of Lineage Species Concept from De Queiroz (2007). Cytogenetic data also provide strong evidence that *Mazama rufa* is composed only by the Paraná cytotype, even considered the close divergence between the Paraná and Carajás cytotypes. They would be in isolation since the reproductive fitness of a potential hybrid could be greatly aggravated by chromosomal imbalance, and thus being practically sterile (Galindo et al., 2021). This is observed in hybrids or populations with the presence of heterozygous tandem fusion, which are considered highly deleterious chromosomal rearrangements, rapidly removed or fixed at meiosis during speciation (King, 1993; Yang et al., 1997; Dobigny et al., 2017; Mudd et al., 2020). Although molecular data indicated the absence of reciprocal monophyly between Paraná and Carajás and suggested both as a single MOTU in the GMYC analyses, this needs to be further explored. The phylogenetic analysis and species delimitation presented here have important limitations, as they assessed only one locus, represented by part of the mitochondrial DNA. Additionally, literature strongly recommends that GMYC results should not be considered alone to guide taxonomic reviews, but should be discussed in the light of other evidences and biological information (Talavera et al., 2013; Tang et al., 2014).

The elucidation of the phylogenetic relationships and species delimitation in the *M. americana* complex depends not only of an advance in molecular analysis, but also an advance in Amazonian populations sampling, which are still underrepresented. Future analyses should include other chromosomal variants associated with the higher diploid number lineage described for this region (Abril et al., 2010). Additionally, the evaluation and nominal description of new species related to the lower diploid number lineage (Juína and Rondônia) also depends on the evaluation of names in the synonymy of *M. americana* in neighboring Amazonian areas such as *Mazama sarae* (Thomas, 1925) described in Bolivia and *Mazama whitelyi* (Gray, 1873) described in Peru.

### Nomenclature Justification

The names and informal descriptions of brocket deer in South America by Azara in “Apuntamientos Para La Historia Natural De Los Quadrúpedos del Paraguay Y Rio De La Plata” based Illiger (1815) formal description. He named the species “Gouazoupitá” (red brocket deer) and gouazoubirá” (brown brocket deer) as *Cervus rufus* and *Cervus simplicicornis,* respectively. These descriptions, according to Allen (1915), were the basis for the nomenclature of the species in the genus and represent a historical landmark. It is necessary, however, to mention that Fischer (1814) also formally described the Azara deer (*Cervus gouazoupita*, the red one and *Cervus gouazoubira*, the brown one). However, Fisher’s work is summarily disregarded in relation to the red brocket deer forest species and the name *Cervus gouazoupita* Fischer, 1814 disappears completely from the literature, never again being mentioned or listed as synonymous in taxonomic reviews of the genus *Mazama*. For example, publications that bring a broad taxonomic organization of the genus, such as that conducted by Lydekker (1898), Allen (1915), Cabrera (1960), and more recently Merino and Rossi (2010), do not even mention *Cervus gouazoupita* Fischer, 1814 and always list *Mazama rufa* (Illiger, 1815) as the valid species. It is important to mention that Illiger makes his work public at the Berlin Academy of Sciences in 1811, the publication of 1815 makes it clear on its back cover that the works described there were presented between 1804 and 1811. Perhaps a previous reading is one of the reasons for his historical preference, given that Illiger’s oral presentation is highlighted as a priority by Allen (1915), and Lydekker (1915) mentions that separate copies were issued in 1811.

The International Code on Zoological Nomenclature is clear that any work published after 1757 [Art. 11.1] must comply with the requirements of providing a public and permanent scientific record and be easily obtainable soon after publication to be considered valid [Art. 8.1], it does not consider public speech or materials issued primarily to participants of scientific meetings [Art. 9.10]. In this regard, a historical recovery would be necessary to prioritize the name *Mazama goazoupita* (Fischer, 1814). However, the priority principle does not aim to change a name already widely used by introducing a new one that is an older synonym or homonym [Art. 23.2]. Given the frequent use and total dominance of the name *Mazama rufa* (Illiger, 1815) by the scientific community, to the detriment of *Mazama gouazoupita* (Fischer, 1814) in the last 200 years, we decided to maintain the use of the first for the sake of clarity and taxonomic stability in the specific case of the red brocket deer of Paraguay-Brazil.

### Distribution and Conservation Aspects

The results indicate that the species is distributed over a wide area, from the south of the continent, in the Atlantic Forest; passing the dry diagonal through the Cerrado and to the southern limit of the Amazon. In addition to occurring in Brazil, it is likely to occur in the Atlantic Forest and Humid Chaco regions of Argentina and Paraguay, as well as possibly in the Dry Forest of Bolivia, on the western edge of the original distribution of *M. americana*. The eastern portion of the red brocket deer distribution, which extends to the coastal Atlantic Forest and the central Cerrado region, needs to be evaluated for the presence of any red brocket deer, whether *M. americana*, *M. bororo* or *M. rufa*.

Despite the wide distribution, this species must be under severe anthropogenic pressure. The forest formations where it occurs in the Atlantic Forest (Araucaria and Interior formations) are the most devastated in the biome, characterized by intense fragmentation (Ribeiro et al., 2009). The conversion of native vegetation and intense fragmentation also impact the Cerrado, which has lost more than 50% of its original cover (Klink and Machado, 2005; Reynolds et al., 2016). The biome also lacks conservation units, with only 3% of its area under strict protection (Françoso et al., 2015). The species was only marginally detected in the Pantanal, an area that did not show suitable habitat. It is important to confirm this information as the presence of a red brocket deer species is known in the area and the region is a refuge for large populations of various mammals, especially deer (Tomas et al., 2010). Finally, the local reality in the southern range of the Amazon where the species was found is of severe deforestation (Santos et al., 2021) and is the most worrying region for *M. rufa* decline.

### Conclusion and Future Directions

The present work demonstrated the revalidation of *Mazama rufa* (Illiger, 1815) as a distinct species, another cryptic species to be separated from the *Mazama americana* complex. It is composed by the former *M. americana* Paraná cytotype and widely distributed throughout South America in the Atlantic Forest, Cerrado, and the south of Amazon. The information presented here should serve as a basis for a detailed assessment of the species extinction risk by the IUCN Red List since it occupies areas of high anthropogenic pressure.

The *Mazama americana* complex begin to be unraveled after a century of divergent taxonomic arrangements. The next steps in the group’s taxonomic review should prioritize: 1) a comprehensive review of museum specimens, accompanied by molecular characterization, and including the analysis of holotypes; 2) sampling and collection of vouchers for cytogenetic characterization with live tissue, especially in the Amazon; 3) a multi-locus molecular analysis, with nuclear markers or population approaches, to better understand the historical and current isolation between close divergence cytotypes.

## Data Availability

The datasets presented in this study can be found in GenBank. Sequences obtained from tissue samples were recorded with the accession numbers MZ488858 to MZ488910 and sequences obtained from fecal samples received the access numbers “MZ521085” to “MZ521234”.

## Supplementary Material

Supplementary Table S1 Animals and Datasets.

Supplementary Table S2 Distribution and Fecal Sampling.

Supplementary Table S3 Morphological Data.

Supplementary Table S4 Molecular Data and Protocols.

Supplementary Table S5 Cytogenetic Data.
