# Supplementary material for: Revalidation of Mazama rufa (Illiger 1815) (Artiodactyla: Cervidae) as a Distinct Species out of the Complex Mazama americana (Erxleben 1777)
Source: Front Genet. 2021 Dec 14;12:742870. doi: 10.3389/fgene.2021.742870 (PMC8712859; doi:10.3389/fgene.2021.742870)
Supplement: Supplementary file 3 [file Table5.DOCX]

***Mazama rufa*- citogenética**

**Material Suplementar**

. A Tabela Y mostra todas as correspondências cromossômicas entre *M. rufa* (MRU) e *B. taurus* (BTA)*;* e entre *M. americana* sensu strictu (MAM) e *B. taurus*.

| **MRU** | **BTA** | **MAM** | **BTA** |
| --- | --- | --- | --- |
| **1** | 1dist, 14, 23 | **1** | 19, 7, 13, 25, 18 |
| **2** | 18, 11, 17 | **2** | 5dist, 28/26, 5prox, 10 |
| **3** | 25, 20, 10 | **3** | 1prox, 20, 3 |
| **4** | 7, 13 | **4** | 1prox, 11, 17 |
| **5** | 3 | **5** | 14, 23 |
| **6** | 4 | **6** | 4 |
| **7** | 2prox | **7** | 2prox |
| **8** | 28, 26 | **8** | 12 |
| **9** | 12 | **9** | 21 |
| **10** | 15 | **10** | 1prox |
| **11** | 21 | **11** | 6prox |
| **12** | 19 | **12** | 9prox |
| **13** | 1prox | **13** | 6dist |
| **14** | 6prox | **14** | 22 |
| **15** | 5prox | **15** | 8prox |
| **16** | 9prox | **16** | 24 |
| **17** | 6dist | **17** | 29 |
| **18** | 22 | **18** | 2dist |
| **19** | 8prox | **19** | 27 |
| **20** | 24 | **20** | 8dist |
| **21** | 29 | **21** | 9dist |
| **22** | 2dist | **X** | X, 16, 15 |
| **23** | 27 | **Y2** | 16, 15 |
| **24** | 8dist |  |  |
| **25** | 9dist |  |  |
| **X** | X, 16, 5dist |  |  |
| **Y_2_** | 16, 5dist |  |  |
