# Supplementary material for: Revalidation of Mazama rufa (Illiger 1815) (Artiodactyla: Cervidae) as a Distinct Species out of the Complex Mazama americana (Erxleben 1777)
Source: Front Genet. 2021 Dec 14;12:742870. doi: 10.3389/fgene.2021.742870 (PMC8712859; doi:10.3389/fgene.2021.742870)
Supplement: Supplementary file 5 [file Table4.DOCX]

**Suplemental Material 04**

**Molecular Data and Protocols**

**PCR Protocolos used for the Tissue Dataset**

The PCR were performed in a final volume of 30ul (27ul of mix and 3ul of 50ng/ul DNA), containing 1x buffer and 1 U of *Taq*  PlatinumTM (Thermo Fisher Scientific), 1,8 mM of MgCl2, 3,6 mM of dNTP, 1 mg/ml of BSA, 0,5 pM of each primer (table 01). Reactions were perfomed using a Bio-Rad C1000 Touch™ Thermal Cycler, under the folowing program: an initial step of 94 ° C for 2 min, followed by 36 cycles of (1) 94 ° C for 1 min, (2) 55 ° C for 30 sec, (3) 72 ° C for 30 sec and a final extension step of 72 ° C for 10 min.

Table 01. Primers used to amplify tissue and blood DNA from brocket deer (*Mazama spp*) samples.

| **Gene** | **Amplicon Size** | **Primer Id** | **Primer Sequence (5'-3')** | **Origin** |
| --- | --- | --- | --- | --- |
| Cytb | 480bp | L14724 | CGAAGCTTGATATGAAAAACCATCGTTG | Irwin et al. (1991) |
|  |  | H15149 | AAACTGCAGCCCCTCAGAATGATATTTGTCCTCA |  |
| Cytb | 660bp | FARH | TCCAATAGTAATAAAGGGGTGTTCA | Duarte et al. (2008) |
|  |  | FARL | CCATGAGGACAAATATCATTCTGAT |  |
| Dloop | 660bp | Thr- L15926 | CAATTCCCCGGTCTTGTAAACC | Vilà et al., (1999) |
|  |  | DL-H16340 | CCTGAAGTAGGAACCAGATG |  |
| ND5 | 690bp | Leu 11775 | GGTGCAACTCCAAATAAAAG | Caparroz et al. (2015) |
|  |  | ND5 12477 | TTTTCCRGTTGCAGCTARTG |  |
| ND5 | 690bp | ND5 12326 | TCTAGCAATAGCATGATTYC | Caparroz et al. (2015) |
|  |  | ND5 12988 | TGTTATTAGGAGGGCTCAGG |  |

**PCR Protocolos used for Fecal Dataset**

The PCR were performed in a final volume of 30ul (25ul of mix and 5ul of fecal DNA), containing 1x buffer and 1,5 U of *Taq*  PlatinumTM (Thermo Fisher Scientific), 2 mM of MgCl2, 3,6 mM of dNTP, 1,3 mg/ml of BSA, 0,5 pM of each primer (table 04). Reactions were perfomed using a Bio-Rad C1000 Touch™ Thermal Cycler, under the folowing touch-down protocol: an initial step of 94 ° C for 2 min, followed by 5 cycles of 94°C-50 sec/ 58°C-50 sec/ 72°C-50 sec; 6 cycles of 94°C-50 sec/ 57°C-45 sec/ 72°C-50 sec; 8 cycles of 94°C-50 sec/ 56°C-40 sec/ 72°C-50 sec; 13 cycles of 94°C-50 sec/ 55°C-30 sec/ 72°C-50 sec; 13 cycles of 94°C-50 sec/ 54°C-30 sec/ 72°C-50 sec and a final extension step of 72 ° C for 10 min.

Table 02. Primers used to amplify fecal DNA from brocket deer (*Mazama spp*) samples.

| **Gene** | **Amplicon Size** | **Primer Id** | **Primer Sequence (5'-3')** | **Origin** |
| --- | --- | --- | --- | --- |
| Cityb | 224 bp | IDMAZ224-L | CATCCGACACAATAACAGCA | González et al 2009 |
|  |  | IDMAZ-H | TCCTACGAATGCTGTGGCTA |  |
| Cityb | 305 bp | Cit305 F | CCATCAGACGCAGACAAA | Leandro, *in prep* |
|  |  | Cit305 R | GCGTTGTTTAGATGTGTGAAG |  |
| Dloop | 305 bp | Dloopb_Fec F | TTGCACTCAACAGCCATATT | present study |
|  |  | Dloopb_Fec R | GACTGCATGGACTTGCTTAT |  |
| ND5 | 249 bp | ND5a_Fec F | TCTCAAACTGACACTGACTTAC | present study |
|  |  | ND5a_Fec R | TGCATCTGTTCGTCCATATC |  |
| ND5 | 249 bp | ND5b_Fec F | CCAACCCTACCTAGCATTTCTC | present study |
|  |  | ND5b_Fec R | TACGACGTATTGGCGGTTTC |  |

**Tissue Dataset Polimorfism**

Table 03. Polimorfism and evolutionary models for the tissue dataset full length aligment and individual mtDNA regions.

| **Dataset** | **n** | **Samples Type** | **Size (bp)** | **Variables Sites (bp)** | **PICs (bp)** | **Partition Finder Best Scheme** | **Models** |
| --- | --- | --- | --- | --- | --- | --- | --- |
| **Full length aligment** | **44** | **Animals** | **2560** | **745** | **518** | **4 subsets** | 1° códon-TRNEF+I+G 2° códon- HKY+I 3° códon-TRN+G Dloop-TRN+I+G |
| ND5 | 44 | Animals | 1128 | 298 | 209 | . | . |
| Cytb | 44 | Animals | 978 | 263 | 180 | . | . |
| Dloop | 44 | Animals | 454 | 184 | 129 | . | . |

**Saturation Test Results And Plot for Tissue Dataset**

Table 04. Test of substitution saturation (Xia et al. 2003; Xia and Lemey 2009) for Animal Dataset Analysis performed on fully resolved sites only. Testing whether the observed Iss is significantly lower than Iss.c. IssSym is Iss.c assuming a symmetrical topology. IssAsym is Iss.c assuming an asymmetrical topology. Two tailed test.

| **NumOTU** | **Iss** | **Iss.cSym** | **T** | **DF** | **p** | **Iss.cAsym** | **T** | **DF** | **p** |
| --- | --- | --- | --- | --- | --- | --- | --- | --- | --- |
| 4 | 0,141 | 0,837 | 57,968 | 814 | 0,00001 | 0,808 | 55,603 | 814 | 0,00001 |
| 8 | 0,148 | 0,816 | 52,801 | 814 | 0,00001 | 0,718 | 45,112 | 814 | 0,00001 |
| 16 | 0,144 | 0,798 | 53,901 | 814 | 0,00001 | 0,621 | 39,308 | 814 | 0,00001 |
| 32 | 0,147 | 0,782 | 53,808 | 814 | 0,00001 | 0,506 | 30,453 | 814 | 0,00001 |

Figure 01. Saturation plot for tissue datset


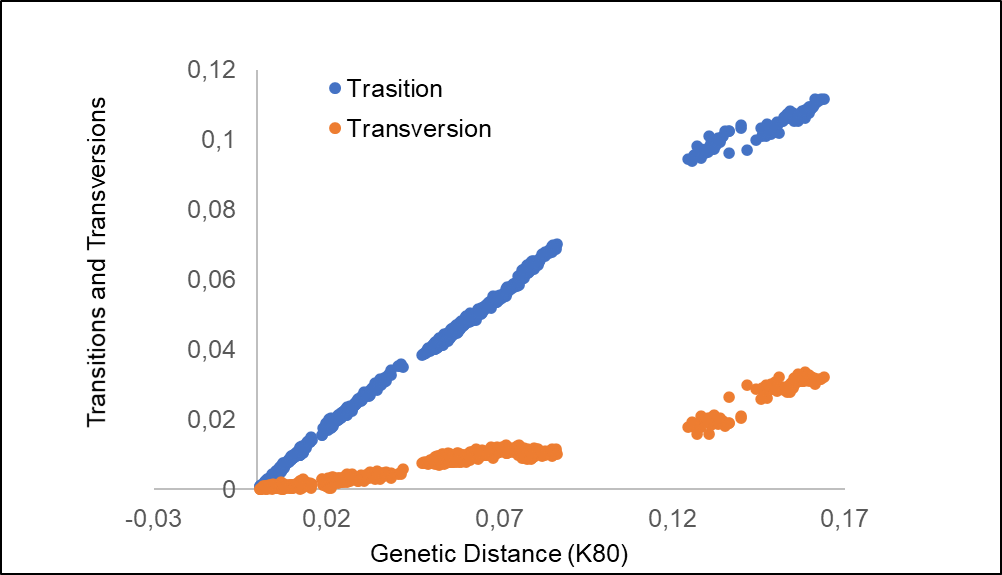


**Saturation Test Results And Plot for Fecal Dataset**

Table 05. Test of substitution saturation (Xia et al. 2003; Xia and Lemey 2009) for Fecal Dataset Analysis performed on fully resolved sites only. Testing whether the observed Iss is significantly lower than Iss.c. IssSym is Iss.c assuming a symmetrical topology. IssAsym is Iss.c assuming an asymmetrical topology. Two tailed test.

| **NumOTU** | **Iss** | **Iss.cSym** | **T** | **DF** | **p** | **Iss.cAsym** | **T** | **DF** | **p** |
| --- | --- | --- | --- | --- | --- | --- | --- | --- | --- |
| 4 | 0,081 | 0,784 | 40,694 | 241 | 0,00001 | 0,756 | 39,043 | 241 | 0,00001 |
| 8 | 0,075 | 0,737 | 37,573 | 241 | 0,00001 | 0,627 | 31,331 | 241 | 0,00001 |
| 16 | 0,079 | 0,69 | 34,578 | 241 | 0,00001 | 0,482 | 22,799 | 241 | 0,00001 |
| 32 | 0,081 | 0,686 | 34,356 | 241 | 0,00001 | 0,356 | 15,626 | 241 | 0,00001 |

Figure 02. Saturation plot for animal datset


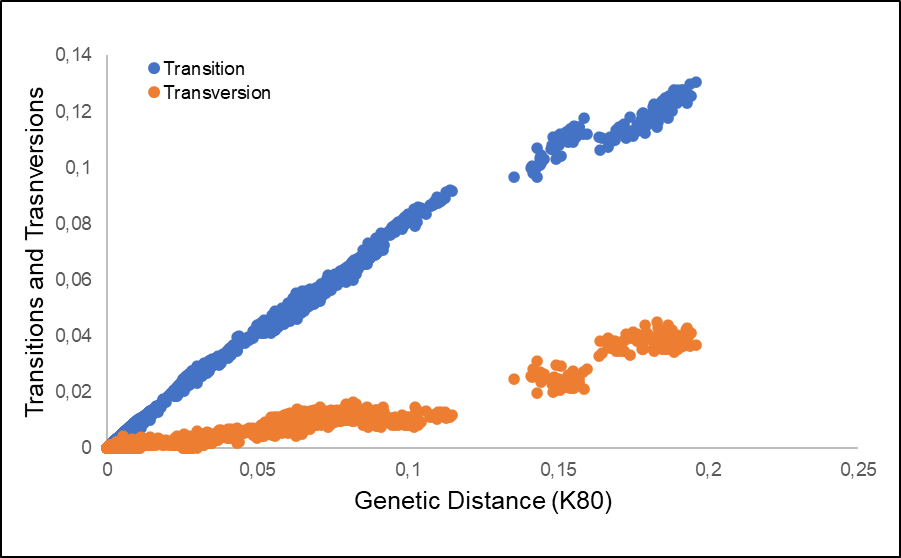


**Partition Finder Analysis for the best Scheme and Model Selection**

Table 06. Partition Finder results for the best partition schemes for tissue dataset.

| **Ranking** | **Scheme Id** | **Sites** | **lnL** | **Parameters** | **Subsets** | **bic** | **delta BIC** |
| --- | --- | --- | --- | --- | --- | --- | --- |
| 1 | 534 | 2560 | -9901.85 | 110 | 4 | 20666 | . |
| 2 | 777 | 2560 | -9872.46 | 120 | 5 | 20686 | 20 |
| 3 | 539 | 2560 | -9888.41 | 117 | 5 | 20695 | 29 |
| 4 | 571 | 2560 | -9895.71 | 117 | 5 | 20709 | 43 |
| 5 | 788 | 2560 | -9859.02 | 127 | 6 | 20714 | 48 |
| 6 | 611 | 2560 | -9920.89 | 113 | 4 | 20728 | 62 |
| 7 | 858 | 2560 | -9866.32 | 127 | 6 | 20729 | 63 |
| 8 | 582 | 2560 | -9882.27 | 124 | 6 | 20737 | 71 |
| 9 | 134 | 2560 | -9925.63 | 113 | 4 | 20738 | 72 |
| 10 | 517 | 2560 | -9926.55 | 114 | 4 | 20747 | 81 |

Table 07. Best partitioning scheme for tissue dataset.

| **Subset** | **Best Model** | **Number of sites** | **Partition names** |
| --- | --- | --- | --- |
| 1 | TRNEF+I+G | 702 | ND5_p1, CytB_p1 |
| 2 | HKY+I | 702 | ND5_p2, CytB_p2 |
| 3 | TRN+G | 702 | ND5_p3, CytB_p3 |
| 4 | TRN+I+G | 454 | Dloop |

Table 08. Partition Finder results for the best partition schemes for fecal dataset.

| **Ranking** | **Scheme Id** | **Sites** | **lnL** | **Parameters** | **Subsets** | **bic** | **delta BIC** |
| --- | --- | --- | --- | --- | --- | --- | --- |
| 1 | 534 | 1103 | -4901.67 | 176 | 4 | 11036 | . |
| 2 | 777 | 1103 | -4898.05 | 178 | 5 | 11043 | 7 |
| 3 | 134 | 1103 | -4915.49 | 174 | 4 | 11049 | 13 |
| 4 | 539 | 1103 | -4896.16 | 181 | 5 | 11060 | 24 |
| 5 | 58 | 1103 | -4933.51 | 171 | 3 | 11065 | 29 |
| 6 | 788 | 1103 | -4892.54 | 183 | 6 | 11067 | 31 |
| 7 | 611 | 1103 | -4922.98 | 175 | 4 | 11071 | 35 |
| 8 | 143 | 1103 | -4909.99 | 179 | 5 | 11074 | 38 |
| 9 | 571 | 1103 | -4900.37 | 182 | 5 | 11075 | 39 |
| 10 | 751 | 1103 | -4904.91 | 181 | 5 | 11077 | 41 |

Table 09. Best partitioning scheme for fecal dataset.

| **Subset** | **Best Model** | **Number of sites** | **Partition names** |
| --- | --- | --- | --- |
| 1 | TRNEF+I+G | 306 | ND5_p1, CytB_p1 |
| 2 | HKY+I | 306 | ND5_p2, CytB_p2 |
| 3 | TRN | 306 | ND5_p3, CytB_p3 |
| 4 | TRN+G | 185 | Dloop |

**Result of GMYC species delimitation for Tissue Dataset**

**Method: single (Figure 03)**

Likelihood of null model: 259.6845

Maximum likelihood of gmyc model: 265.9788

Likelihood ratio: 12.5886

Result of lr test: 0.001846804 (significant)

Number of ml clusters: 8

Confidence interval: 2-11

Number of ml entities: 9

Confidence interval: 2-19

Threshold time: -0.009657144

**Method: multiple**

Likelihood of null model: 264.2216

Maximum likelihood of gmyc model: 266.5656

Likelihood ratio: 4.688157

Result of lr test: 0.09593555 non significant

Number of ml clusters: 7

Confidence interval: 4-8


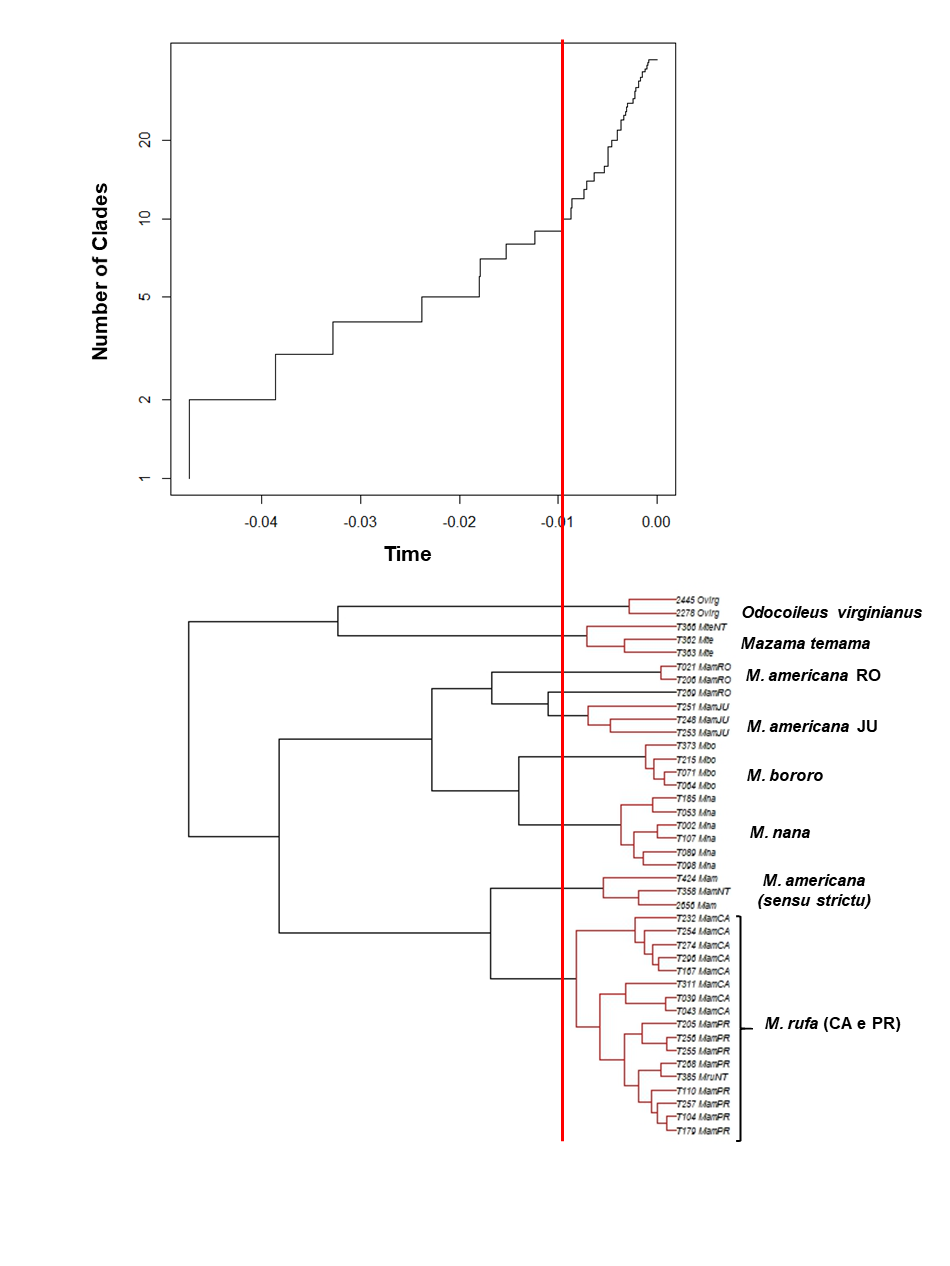


Figure 03. Result of GMYC single threshold method species delimitation for animal dataset.

**Fecal Dataset Species Identification**


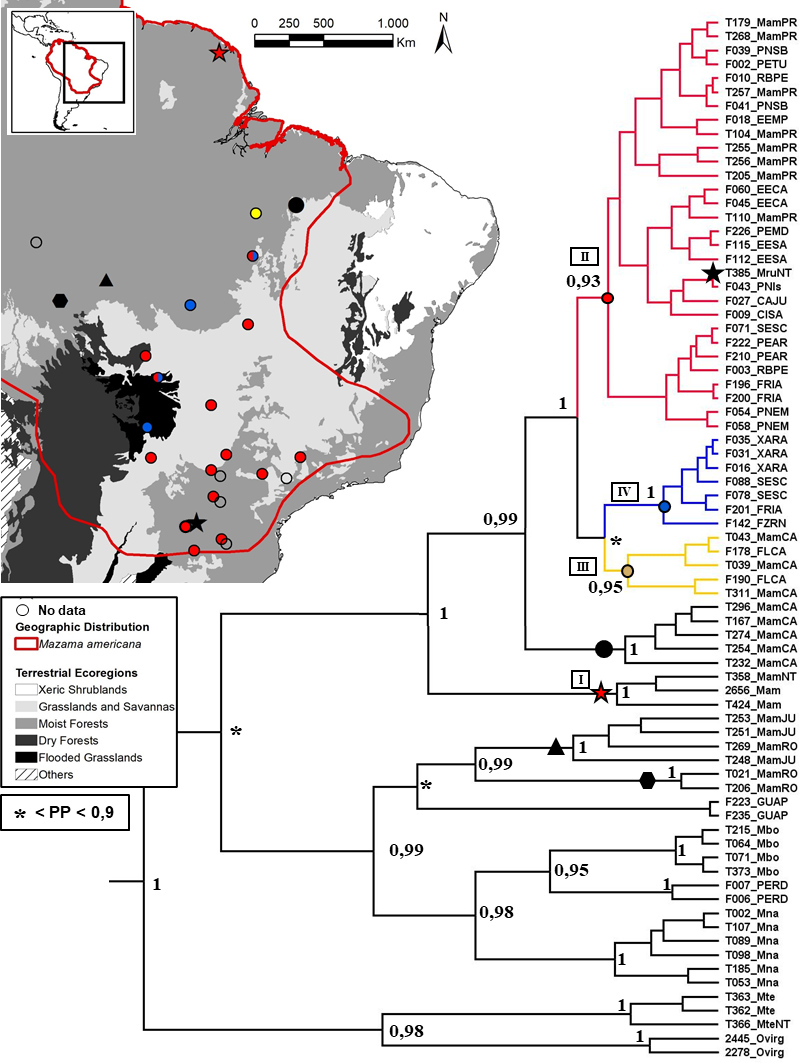


Figure 04-Summary of the phylogenetic inference results to identify *Mazama rufa* (Illiger, 1815) (Odocoileini: Capreolinae: Cervidae) fecal samples indicated as red dots in the map and red clade in the tree. The figure shows a map with the fecal sampling sites distribution towards South America terrestrial ecoregios and an ultrametric tree representing the phylogenetic hypothesis generated by Bayesian Inference by the BEAST package. The support values of the clades are represented by the posterior probability (PP 0-1). Numbers I to IV identify clades highlighted in the text. Sequences identified with T### represent sampled animals and sequences identified by #### are those obtained from GenBank, * indicates neotypes.
